# Diagnostic Performance of MRI to Differentiate Uterine Leiomyosarcoma from Benign Leiomyoma: A Meta-Analysis

**DOI:** 10.5334/jbsr.2275

**Published:** 2020-11-24

**Authors:** Mayur Virarkar, Radwan Diab, Sarah Palmquist, Roland Bassett, Priya Bhosale

**Affiliations:** 1 Department of Diagnostic Radiology, The University of Texas MD Anderson Cancer Center, Houston, Texas, US; 2 Department of Biostatistics, The University of Texas MD Anderson Cancer Center, Houston, Texas, US

**Keywords:** MRI, ADC, systematic review, meta-analysis, leiomyosarcoma and leiomyoma

## Abstract

**Purpose:**

To perform a meta-analysis comparing the diagnostic performance of increased signal intensity on T1- and T2-weighted magnetic resonance images and apparent diffusion coefficient (ADC) values in differentiating uterine leiomyosarcoma (LMS) from benign leiomyoma (LM).

**Methods:**

A systematic literature search for original studies was performed using PubMed/MEDLINE, the Cochrane Library, Embase, and Web of Science. Data necessary for the meta-analysis was extracted from the selected articles and analyzed.

**Results:**

Eight studies with 795 patients met our predefined inclusion criteria and were included in the analysis. Increased signal on T1-weighted imaging had a pooled sensitivity of 56.8% (95% CI: 20%–87.4%) for LMS (n = 60) which was significantly higher than 7.6% (95% CI: 2.2%–22.7%) for LM (n = 1272) (*p* = 0.0094). Increased signal analysis on T2-weighted imaging had a pooled sensitivities of 93.2% and 93.2% (95% CI: 45.7%–99.6% and 42.9%–99.6%) for LMS (n = 90), which were not significantly different from the 54.5% and 53.9% (95% CI: 33.6%–74%, 32%–74%) for LM (n = 215) (*p* = 0.102 and 0.112). On ADC value analysis, LMS (n = 43) had a weighted mean and standard deviation of 0.896 ± 0.19 10^–3^ mm^2^/s, 0.929 ± 0.182 10^–3^ mm^2^/s, which were significantly lower from 1.258 ± 0.303 10^–3^ mm^2^/s, 1.304 ± 0.303 10^–3^ mm^2^/s for LM (n = 159) (*p* = < 0.0001, < 0.0001).

**Conclusion:**

Our meta-analysis demonstrated that high signal intensity on T1-weighted images and low ADC values can accurately differentiate LMS from LM. Although, LMS had a higher pooled sensitivity for T2-weighted increased signal intensity compared to LM, there was no statistical significance.

## Introduction

**Figure 1:**
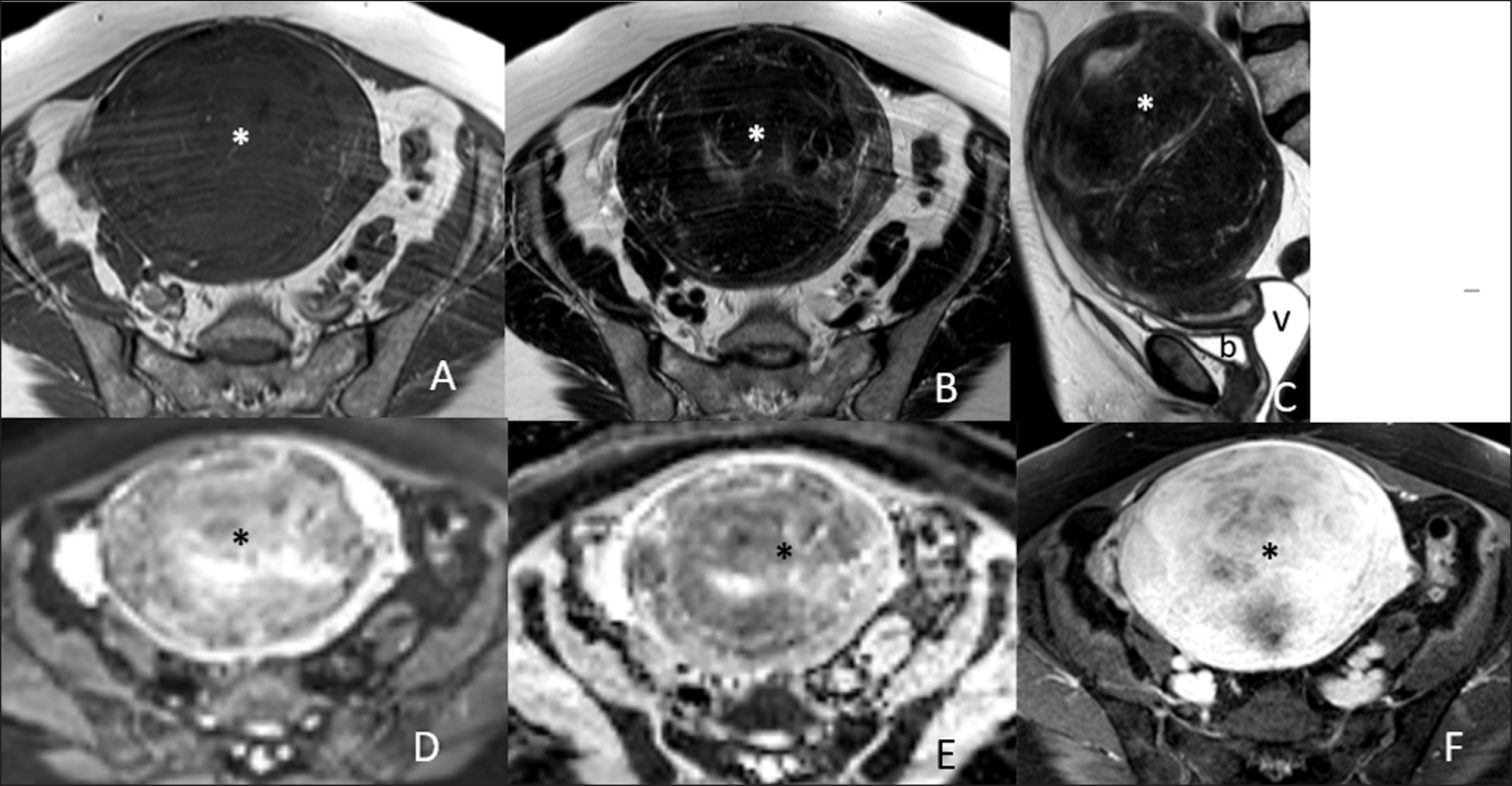
60-year-old woman with LM. **(A)** Axial T1WI, **(B)** axial T2WI, **(C)** axial diffusion and **(D)** ADC maps MRI show a large, heterogeneously T1 and T2 low signal intensity LM (asterisk).

**Figure 2:**
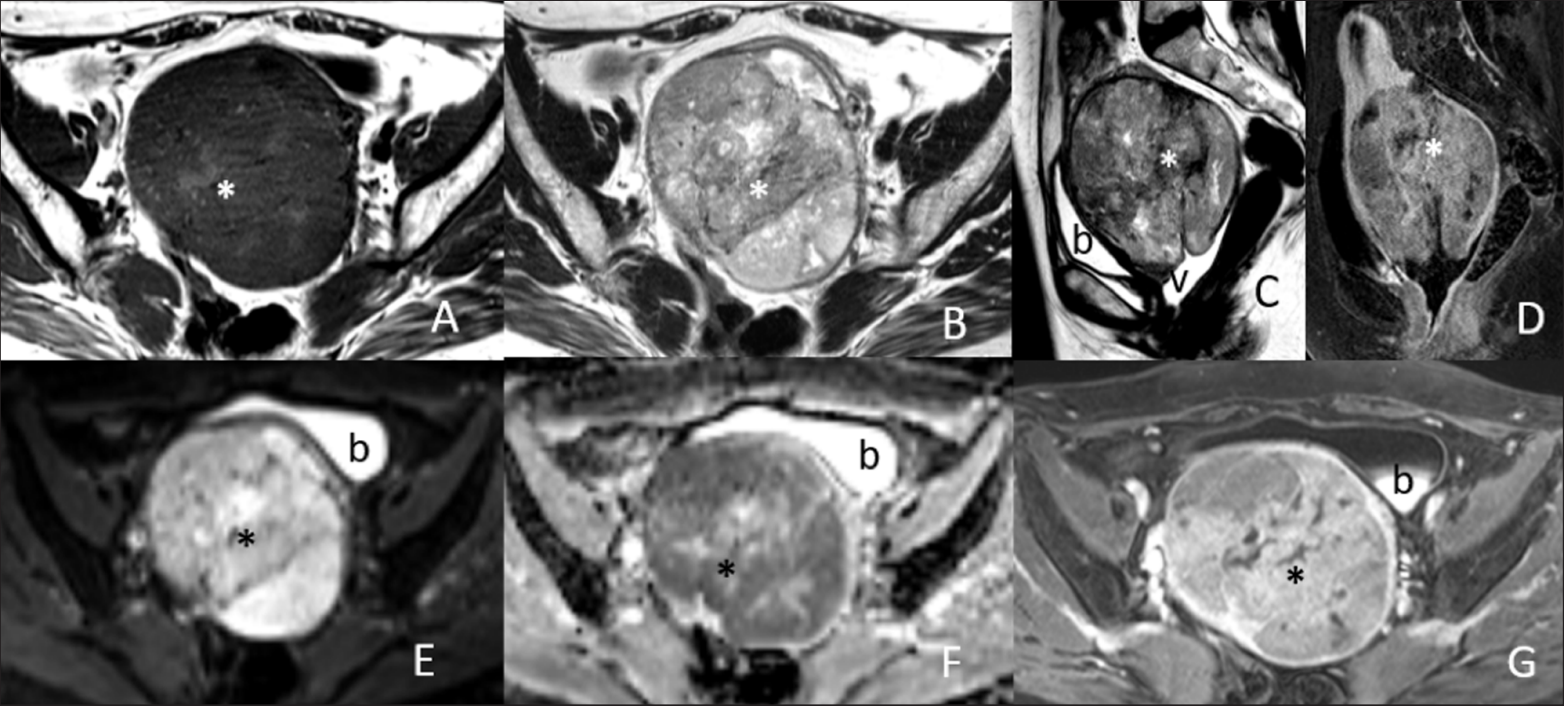
65-year-old woman with LMS. **(A)** Axial T1WI, **(B)** axial T2WI, **(C)** axial diffusion and **(D)** ADC maps MRI show a large, heterogeneously predominantly T1 low signal and T2 high signal LM (asterisk). Bladder (b).

Uterine leiomyomas (LMs) are the most common benign smooth muscle tumors of the uterus; they occur in perimenopausal as well as reproductive-age women (Figure 1)[1]. On the malignant spectrum, uterine sarcomas tend to occur in older patients and account for 3%–7% of all uterine malignancies. Leiomyosarcomas (LMS) (Figure 2) are the most common uterine sarcomas, with an estimated annual incidence of 0.5–7 per 100,000 women [2]. Pre-operative diagnosis of LMS is often challenging. Several studies have attempted to identify magnetic resonance imaging (MRI) characteristics that can be used to preoperatively distinguish LMS from LM. Some imaging features, such as ill-defined margins, increased signal intensity on T1WI and T2WI, hemorrhage, central necrosis, diffusion-weighted imaging characteristics, and texture analysis histogram metrics, have shown promising results, but there is a lack of consensus in the current literature [3,4,5,6,7,8,9,10,11,12].

We performed a meta-analysis to identify the MRI features that effectively distinguish LMS from LM.

## Materials and Methods

We followed the Preferred Reporting Items for Systematic Reviews (PRISMA) guidelines in this study. All available literature published through December 2019 was searched using PubMed/MEDLINE, Embase, and the Cochrane Database of Systemic Reviews. The databases were comprehensively searched using the following keywords:

- ‘MRI,’ ‘MR,’ or ‘magnetic resonance imaging’;
- ‘leiomyoma’ or ‘fibroid;’ and
- leiomyosarcoma.’

The reference lists of all retrieved studies were scrutinized to identify additional articles to supplement the search results.

### Inclusion Criteria

The inclusion criteria were as follows: 1) studies of the diagnostic accuracy of MRI to differentiate between LMS and LM, 2) human studies, 3) complete original publications, and 4) studies with a histopathologic analysis or imaging features as the standard of reference.

### Exclusion Criteria

The exclusion criteria were as follows: 1) studies not published in English; 2) review articles, meeting abstracts, letters, case reports, and articles without sufficient data; and 3) diagnostic techniques other than MRI.

### Study Quality Assessment

A single reviewer (M.V.) assessed the quality of all eligible studies using the current Quality Assessment of Diagnostic Accuracy Studies (QUADAS)-2 tool. This tool includes four major domains: patient selection, index test, reference standard, and flow and timing. These domains were then further assessed on the basis of the risk of bias, and their applicability was rated as ‘high,’ ‘low,’ or ‘unclear.’ A second reviewer (P.B.) assessed the accuracy of this assessment.

### Data Extraction

The following data were extracted from each study: 1) title, author or authors, country in which the study was performed, year published, study type, and MRI scanner; 2) number of patients; 3) study objective; 4) primary findings; and 5) statistical data for analysis.

### Diagnostic Performance Analysis

To reduce clinical (pretest probability of malignancy) and methodologic heterogeneity, our primary diagnostic performance analysis included increased signal intensity on T1WI and T2WI and the ADC values of LM and LMS.

### Standard of Reference

The standard of reference was histologic confirmation of the lesion (obtained during surgery or biopsy). In the meta-analysis, eight studies used histologic confirmation as the only standard of reference. In the Ando et al. study, histologic confirmation and imaging features served as the reference standard [3].

### Statistical Analysis

Data on T1WI or T2WI signal intensity were collected from eight selected studies and included the true number of patients with disease (true positive) and the number detected on T1WI or T2WI. Analyses were performed separately for LMS and LM data, calculating the sensitivities of increased signal intensity on T1WI and T2WI. Sensitivity was estimated with the available study-level data from a random effects model using the DerSimonian and Laird approach [13]. ADC values were compared between LMS and LM. Means and standard deviations were obtained for each study, and an overall pooled mean and standard deviation (SD) were calculated. A t-test was performed using these values to compare LMS and LM. All statistical analyses were performed using R software version 3.6.1. All statistical tests used a significance level of 5%. There were no adjustments required for multiple testing.

## Results

### Study Selection and Description

**Figure 3:**
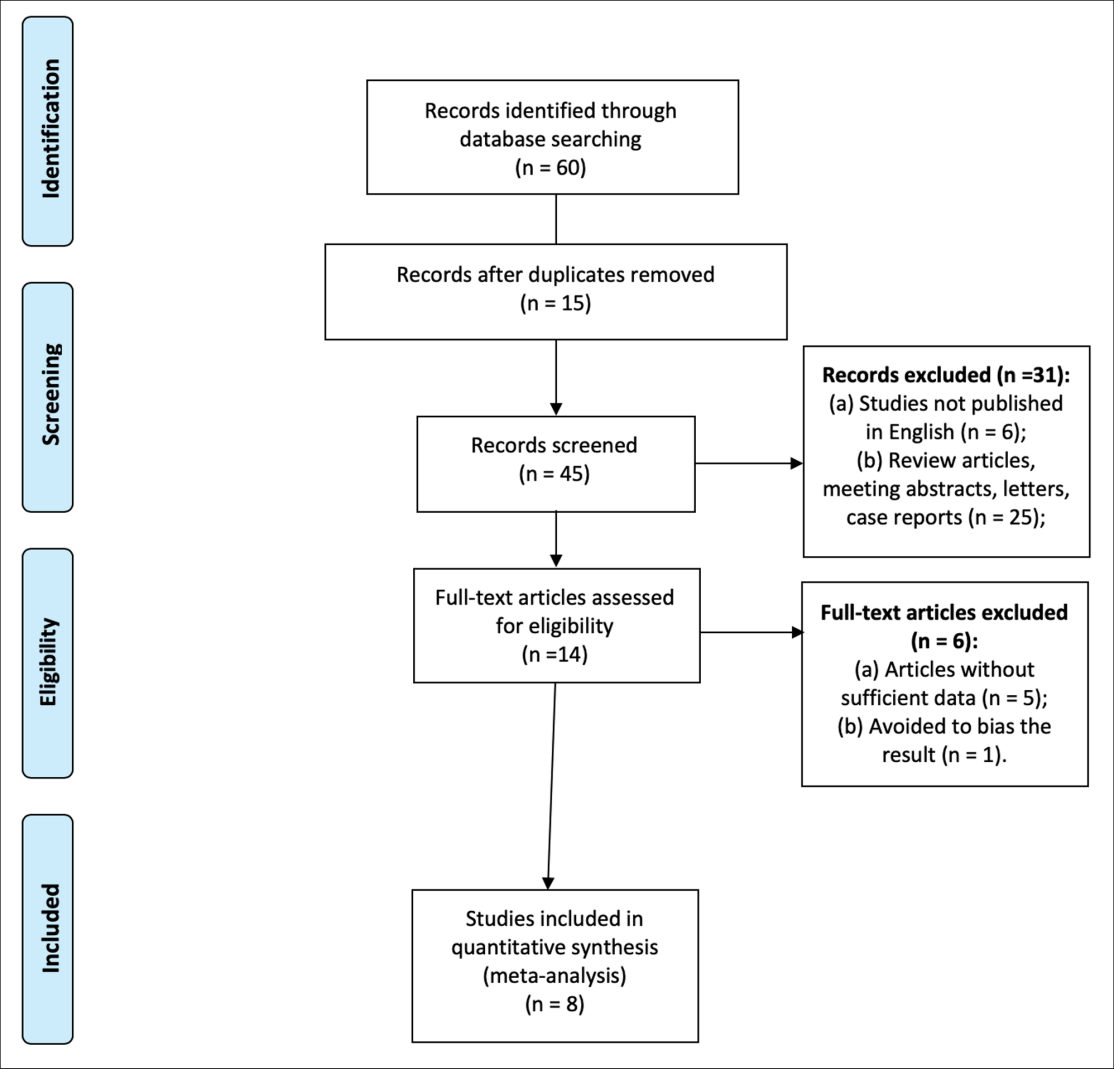
Preferred Reporting Items for Systematic Reviews and Meta-Analyses (PRISMA) flow diagram.

The initial database search yielded 60 articles. After we removed all duplicate studies, 45 remained. We reviewed the titles and abstracts to identify articles that were irrelevant to our analysis or were reviews, case reports, letters, or editorials and excluded 31, leaving 14 potentially eligible articles. Of these, five lacked sufficient data and were excluded, leaving a total of 9 (Table 1). A flow diagram of the study selection procedure is shown in Figure 3.

**Table 1:** Characteristics of the Selected Studies in the Meta-Analysis.

Study	Year	Country	Study type	Objective	Subjects	MRI SI and ADC value	MRI scanner	Primary findings

Sato et al. [8]	2014	Japan	R	Determine the clinical utility of DWI and ADC in differentiating between LM and LMS.	10 LMS (5 patients), 83 LM (76 patients)	ROI used to measure the ADC values was as large as possible within the solid tumoral component, not contain necrotic cysts as much as possible by referring to the T2WI, and not contain high-intensity areas on the T1WI. If several ADC values were measured, the lowest value was adopted.	1.5-T MRI (Excelart Vantage version 8.02; Toshiba Medical Systems Corp., Ouattara, Tochigi Prefecture, Japan) using an eight-channel phased-array coil.	Mean ADC value for the 10 LMS was 0.791 ± 0.145, significantly lower than that of the 41 LM nodules that presented with intermediate-intensity areas (1.472 ± 0.285, *p* < 0.001) and nine with high-intensity areas (1.100 ± 0.343, *p* = 0.03).
Lahkman et al. [4]	2016	USA	R	Identify the MR features that distinguish LMS from atypical leiomyoma (ALM).	22 ALM, 19 LMS (41 patients)	SI of relative to the outer myometrium on T2WI.	1.5-T MRI (GE Medical Systems, Milwaukee, WI) using pelvic phased-array coils.	Four qualitative MRI features most strongly associated with LMS were nodular borders, hemorrhage, “T2 dark” areas, and central unenhanced areas (*p* ≤ 0.0001). Sixteen texture features differed significantly between LMS and ALM (*p* < 0.001–0.036).
Ando et al. [3]	2018	Japan	R	Evaluate the differences between uterine LMS and LM.	1118 LM, 14 LMS, 5 STUMP (509 patients)	T1WI was visually defined as SI higher than that in the skeletal muscles at the same level. Second, if the reviewers confirmed the presence of hyperintensity on T1WI within the tumor, the presence of fat tissue on fat suppressed T1WI was also evaluated.	1.5-T MRI (Intera Achieva 1.5 T Pulsar; Philips Medical Systems, Best, Netherlands) or 3-T MRI (Achieva Quasar Dual 3 T; Philips Medical Systems, Best, Netherlands).	T1WO hyperintense areas were observed in 11 of 14 (78.6%) LMS, 0 of 5 (0%) STUMP, and 15 of 1118 (1.3%) LMs. T1 hyperintense areas within LM showed more homogeneity, better demarcation, a smaller occupying rate, and higher SI than did T1 hyperintense areas within LMS.
Li et al. [5]	2017	China	R	Evaluate the diagnostic efficiency of DWI in separating LMS from LM.	26 LM, 16 LMS (42 patients)	SI of the solid components on T1WI and T2WI (hypointensity, isointensity, and hyperintensity, respectively, contrasting with the SI of outer myometrium). ADC value of the solid component; the measurement was performed on an ADC map with a single ROI.	1.5-T MRI (Avanto or Espree; Siemens, Erlangen, Germany) with a phased-array abdominal coil.	Mean ADC value in LMS (0.81–0.9) was significantly lower than that in LM (1.22–1.50) (*p* < 0.001).
Valdes-Devesa et al. [11]	2019	Spain	R	Determine the utility of DWI in differentiating LM and sarcomas.	17 LM, 6 LMS, 4 other sarcoma (27 patients)	High SI on T2WI.	3-T MRI (GE Medical Systems, Milwaukee, WI, USA).	Calculated ADC values were ^3^ 1.00 for benign tumors (n = 17) and < 1.00 for sarcomas (n = 10) without overlap.
Tanaka et al [10]	2004	Japan	R	Use MRI to characterize uterine smooth muscle tumors.	12 LM, 9 LMS, 3 STUMP (24 patients)	High SI on T1WI when it showed higher signal than fatty bone marrow in the pubic symphysis. High SI on T2WI when the SI of the mass was higher than that of the outer myometrium, and the areas of high signal accounted for more than 50% of the mass, were classified as hyperintense on T2WI.	1.5-T MRI (Signa, GE Medical Systems, Milwaukee, WI; and Gyroscan ACS-NT, Best, Netherlands) and a 1.0-T (Magnetom Harmony; Siemens, Erlangen, Germany) with a phased-array body and body coil.	50% hyperintensity on T2WI and the presence of any small hyperintense foci on T1WI with unenhanced pockets were considered MRI suggestive of STUMP and LMS.
Tamai et al. [9]	2007	Japan	R	Evaluate the utility of DWI in differentiating uterine sarcomas from LM.	51 LM, 5 LMS, 2 endometrial stromal sarcoma (43 patients)	High SI on T1WI when it showed higher signal than fatty bone marrow in the pubic symphysis. Focal areas of high signal were grouped as high on T1WI. High SI on T2WI when the areas of higher signal compared with that of the outer myometrium accounted for more than 50% of the tumor. Necrosis or cystic spaces were not involved.	1.5-T MRI (Symphony; Siemens, Erlangen, Germany) using a six-channel phased-array coil.	Mean ADC value of sarcomas was 1.17 ± 0.15, which was lower than those of the normal myometrium (1.62 ± 0.11) and degenerated LM (1.70 ± 0.11) with no overlap; however, they were overlapped with those of ordinary LM and cellular LM.
Lin et al. [6]	2016	Taiwan	P	Compare the diagnostic accuracy of CE-MRI and DWI between uterine LMS/STUMP and LM.	25 LM, 6 LMS, 2 STUMP (33 patients)	Manually drawn ROIs within nonnecrotic tumors on T2WI axial images, with the ROIs copied from T2WIs onto the ADC map.	3.0-T MRI (Trio Tim, Siemens Medical Systems, Erlangen, Germany) using phased-array body coils.	CE-MRI yielded a significantly superior diagnostic accuracy (0.94 vs. 0.52) and higher specificity (0.96 vs. 0.36) than did DWI (*p* < 0.05 for both); it had a similarly high sensitivity (0.88 vs. 1.00) for prospective differentiation between uterine LMS or STUMP and LM.

R: Retrospective, P: Prospective, DWI: Diffusion Weighted Images, ADC: apparent diffusion coefficient, STUMP: Uterine smooth muscle tumor of uncertain malignant potential, ROI: region of interest, T1WI: T1 weighted images, T2WI: T2 weighted images, CE-MRI: Contrast Enhancing Magnetic Resonance Imaging, SI: Signal Intensity.

### QUADAS-2 Assessment

The results of the Quality Assessment of Diagnostic Accuracy Studies-2 (QUADAS-2) are presented in Table 2. We found a low risk of bias with regard to participant selection, except in two studies. In the study by Li et al., patients with LM with red degeneration and lipoleiomyoma were excluded [5]. In the Addo et al. study, only those with degenerated LM were included [3]. With respect to the reference standard, we believe that there was a low risk of bias in the majority of studies because all, but one study used histopathologic confirmation of diagnosis as the reference standard. In the study by Addo et al., histologic confirmation and imaging features served as the reference standard [3]. In the index test domain, studies had a low risk of bias because of blinding of the diagnosis to the reference test. On the flow and timing domain, some studies could not be evaluated in terms of the risk of bias, as they did not provide the interval between the index test and the reference standard [3,4,8,11].

**Table 2:** Assessment of the Quality of the Selected Studies Using QUADAS-2.

Study	Risk of bias				Applicability concerns		

	Patient selection	Index test	Reference standard	Flow and timing	Patient selection	Index test	Reference standard

Sato et al. [8]	☺	☺	☺	?	☺	☺	☺
Lahkman et al. [4]	☺	☺	☺	?	☺	☺	☺
Ando et al. [19]	☹	☺	☹	?	☹	☺	☹
Li et al. [5]	☹	☺	☺	☺	☹	☺	☺
Valdes-Devesa et al. [11]	☺	☺	☺	?	☺	☺	☺
Tanaka et al. [10]	☺	☺	☺	☺	☺	☺	☺
Tamai et al. [9]	☺	☺	☺	☺	☺	☺	☺
Lin et al. [6]	☺	☺	☺	☺	☺	☺	☺

☺ Low risk      ☹ High risk       ? Unclear risk.

### Diagnostic Performance

**Figure 4:**
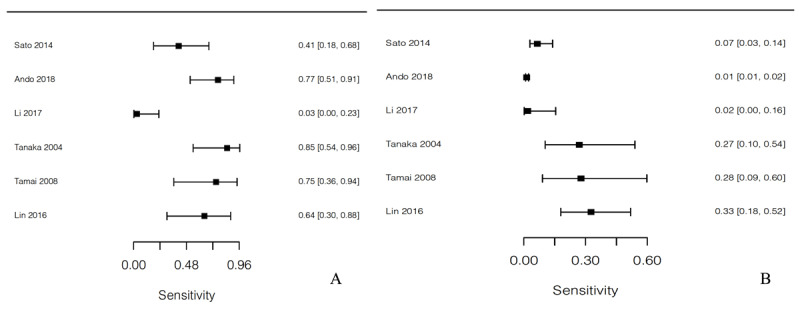
Forest plot of studies in the meta-analysis showing sensitivity of high signal intensity on T1WI for **(A)** LMS and **(B)** LM.

**Figure 5:**
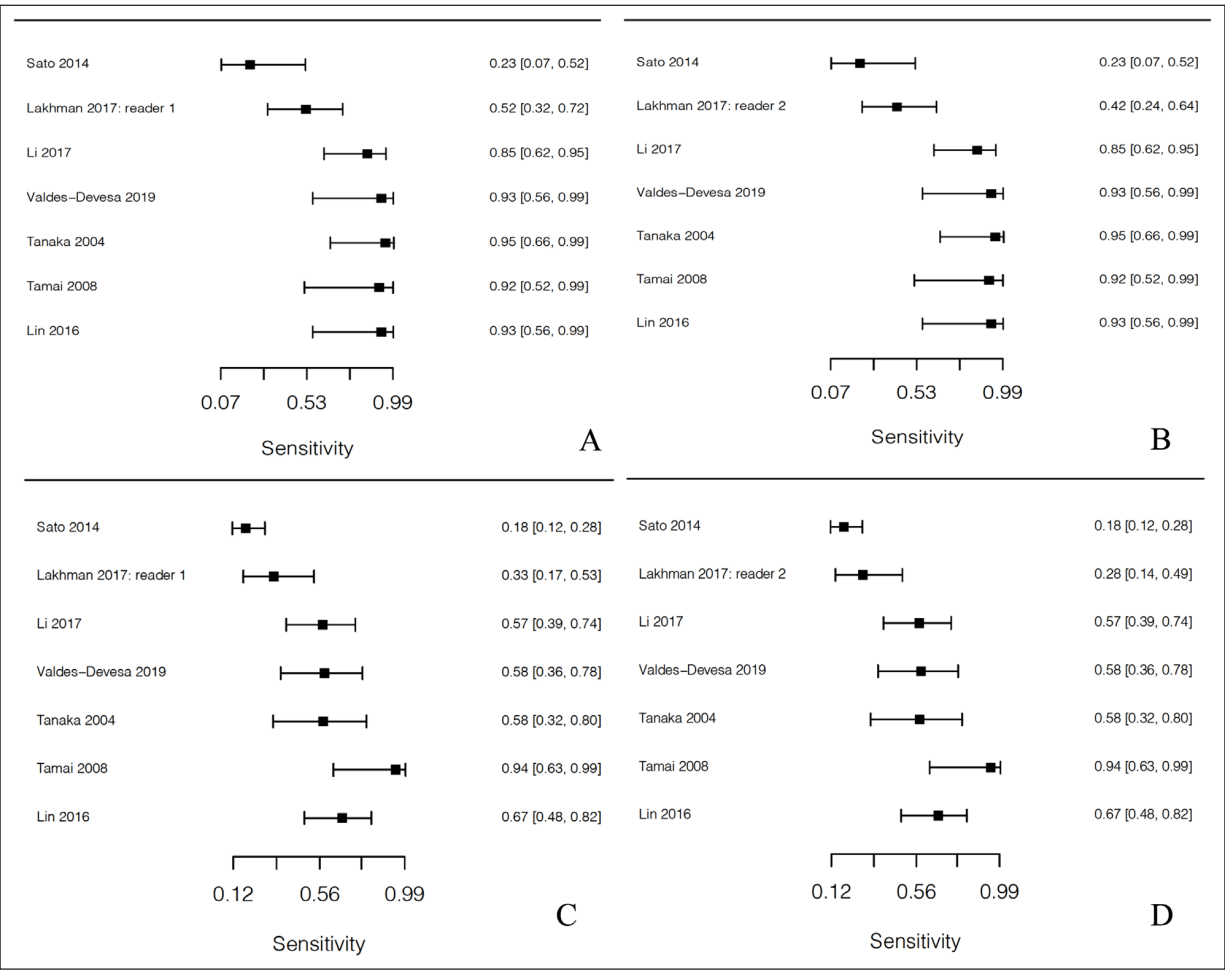
Forest plot of studies in the meta-analysis showing sensitivity for high signal intensity on T2WI for LMS for reader 1 **(A)**, reader 2 **(B)** and sensitivity for high signal intensity on T2WI for LM for reader 1 **(C)**, reader 2 **(D)**.

We performed a T1WI high signal intensity analysis and found that its pooled sensitivity for LMS (56.8%) was significantly higher than for LM (7.6%) (*p* = 0.0094). The T2WI high signal intensity analysis, considering only reader 1 and only reader 2 in the Lakhman et al. article [4], revealed that the sensitivity for LMS (93.2% and 93.2%) was not significantly different from that for LM (54.5% and 53.9%) (*p* = 0.102 and 0.112). The ADC value analysis (methods 1 and 2 in Li et al. [5]) revealed a weighted mean and SD for LMS (0.896 ± 0.19 × 10^–3^ mm^2^/s, 0.929 ± 0.182 × 10^–3^ mm^2^/s) that were significantly lower than were those for LM (1.258 ± 0.303 × 10^–3^ mm^2^/s, 1.304 ± 0.303 × 10^–3^ mm^2^/s) (*p* < 0.0001, < 0.0001) (Tables 3, Figures 4 and 5).

**Table 3:** Meta-analysis data of selected studies for LMS and LM.

No	Study	LMS				LM				LMS			LM		

		T1WI		T2WI		T1WI		T2W1		ADC			ADC		

		TP	FN	TP	FN	TP	FN	TP	FN	Mean	SD	n	Mean	SD	n

1	Sato et al. [8]	4	6	2	8	5	78	15	68	0.791	0.145	10	1.234	0.346	83
2	Lahkman et al. [4]														
	Reader 1	–	–	10	9	–	–	7	15	–	–	–	–	–	–
	Reader 2	–	–	8	11	–	–	6	16	–	–	–	–	–	–
3	Ando et al. [3]	11	3	–	–	15	1103	–	–	–	–	–	–	–	–
4	Li et al. [5]														
	Reader 1	0	16	14	2	0	26	15	11	0.810	0.140	16	1.220	0.220	26
	Reader 2									0.900	0.110	16	1.500	0.220	26
5	Valdes-Devesa et al. [11]	–	–	6	0	–	–	10	7	–	–	–	–	–	–
6	Tanaka et al. [10]	8	1	9	0	3	9	7	5	–	–	–	–	–	–
7	Tamai et al. [9]	4	1	5	0	2	6	8	0	1.240	0.045	5	1.569	0.260	8
8	Lin et al. [6]	4	2	6	0	8	17	17	8	1.050	0.410	6	1.200	0.270	25

TP, true positive, FN, false negative. n: Number of lesions.

## Discussion

We found that high signal intensity on T1WI and low ADC values can be used to differentiate LMS from LM. LMS had higher pooled sensitivity on T2WI than did LM, but this result was not statistically significant.

On MRI, LMs usually present as solitary or multifocal, well-defined masses of variable size, with low signal intensities on T1WI and T2WI. However, LM with red degeneration and lipoleiomyoma demonstrates high signal intensity on T1WI, and cystic and degenerative LM often show high signal intensity on T2WI [1]. LMS usually presents as a heterogeneous and poorly demarcated mass, with variable appearance on T1WI. It may show low or intermediate signal intensity on T1WI, similar to LM, but it frequently demonstrates areas of high signal intensity, corresponding to hemorrhage or necrosis. On T2WI, LMS shows an intermediate to high signal because of its high cellularity. In addition to the imaging discrepancies between LMS and LM, there are discordant results in the literature on the diagnostic accuracy of high signal intensity on T1WI and T2WI [14].

An earlier systemic review by Kaganov et al. reported that there was a significant relationship between T1WI and T2WI signal intensities on and tumor pathologic characteristics (*p* < 0.05) but not between ADC values and tumor pathologic characteristics (*p* = 0.18) [15]. The decision tree diagram indicates that low signal intensity on T1WI and T2WI was most commonly associated with LM, whereas high signal intensity was a good indicator of LMS. Our meta-analysis has the advantage of a larger patient cohort. Tanaka et al. reported that a more than 50% high signal intensity on intralesional T2WI, focal high signal on T1WI, and unenhancing regions can be used to distinguish LMS from LM [10]. High signal intensity on T1WI and T2WI had 72.7% sensitivity, 100% specificity, 100% positive predictive value, 80% negative predictive value, and 87.0% overall accuracy. A prospective study conducted by Lin et al. reported that the sensitivity of T2WI was significantly higher than that of T1WI (0.88 vs 0.63, *p* < 0.05) [6]. They also found that the specificity (0.96) and AUC (0.92) of contrast-enhanced MRI were significantly higher than those of diffusion-weighed imaging, T2WI, or T1WI. Valdes-Devesa et al. reported that a high or inhomogeneous signal on T2WI and poorly defined borders were significantly more common in sarcomas (LMS, carcinosarcoma, and endometrial stromal sarcoma) than in LMS [11].

Contrary to the results of our meta-analysis, Ando et al. reported that LMs had more homogenous high signal intensity on T1WI, were well-defined, had more frequently T2 hypointense rims, had greater signal intensity ratios of high signals on T1WI (high signal intensity area-to-skeletal muscle signal intensity ratio), and had lower high signal occupying rates on T1WI (high signal intensity on T1-to-whole tumor square measure ratio) than did LMS (*p* < 0.05) [3]. Lipoleiomyomas and LM with red degeneration exhibit a high signal on T1WI, which is why they were excluded from the study as potential confounding factors. Lakhman et al. reported that there was no significant difference in the T2WI signal intensity in LMS and LM for the two readers in their study (*p* = 0.034, 0.017), with a substantial interpretation agreement (k = 0.80, 95% CI, 0.64–0.96) [4]. Li et al. found that there was no significant difference in the heterogeneous high signal on T2WI between LMS (88%) and degenerated LM (58%) [5].

Some studies have reported inconclusive results for T1WI and T2WI features. One study reported that high signal intensity on T1WI and T2WI was less common in both LMS and LM (40% vs 6%, 20% vs 18%, respectively) [8]. In a study by Tamai et al., 80% (4 of 5) of LMS cases had high intensity on T1WI compared with 100% on T2WI [9]. The LM cases were subdivided into cellular, degenerated, and ordinary. All cellular LMs had low signal intensity on T1WI and high on T2WI. All ordinary LMs had low signal intensity on T1WI and T2WI; degenerated LMs had high signal intensity on T2WI.

Diffusion-weighted imaging is based on the diffusion motion of water molecules. It is widely used to distinguish between malignant and benign tumors by measuring the ADC value [16]. In general, a low ADC value is correlated with malignant lesions, as their higher cellularity and total nuclear area restrict water diffusion [17,18]. Previous studies have shown that ADC values can help distinguish uterine sarcoma from LM [5,6,7,8,9,11,12]. However, an overlapping of the ADC value between ordinary LMs and LMS has also been mentioned [6, 9]. Our findings are in consensus with those of most previous studies, as we found that the weighted mean and SD for LMS (0.896 ± 0.19, 0.929 ± 0.182) were significantly lower than were those of LM (1.258 ± 0.303, 1.304 ± 0.303) (*p* < 0.0001, < 0.0001).

Our meta-analysis results are supported by those of Sato et al. study they reported that the mean ADC value of LMS (0.791 ± 0.145) was significantly lower than that of LM with intermediate (1.472 ± 0.285) and high intensity (1.100 ± 0.343) (*p* < 0.05) [8]. Li et al. reported that the ADC value of LMS (0.81 ± 0.14 and 0.90 ± 0.11) was significantly lower than that of degenerated LM (1.22 ± 0.22 and 1.50 ± 0.22) (*p* < 0.001, < 0.001, respectively) [5], and Valdes-Devesa et al. reported that the postoperatively calculated ADC values in their study were significantly lower in the sarcoma group (0.84 ± 0.09) than in the LM group (1.37 ± 0.23) [11].

Tamai et al. reported that the ADC values of uterine sarcomas (1.17 ± 0.15) were lower than were those of the normal myometrium (1.62 ± 0.11) and degenerated LM (1.70 ± 0.11), with no overlap [9]. The ADC values overlapped with those of ordinary LM (0.88 ± 0.27) and cellular LM (1.19 ± 0.18). Similarly, in the study by Lin et al., the ADC value of the combined LMS and Uterine smooth muscle tumor of uncertain malignant potential (STUMP) (median, 0.89; range, 0.74–1.85) was significantly lower than that of LM (median, 1.19; range, 0.70–2.04; *p* < 0.05) [6]. Nonetheless, the ADC values overlapped among LMS (mean ± SD, 1.05 ± 0.41), STUMP (0.92 ± 0.13), and LM, including cellular (1.43 ± 0.58), infarcted (1.23 ± 0.50), degenerated (1.17 ± 0.17), and ordinary LM (1.14 ± 0.16).

Our study had some limitations. First, our primary analysis was limited because of the small number of studies (n = 8) included in the meta-analysis. Second, combining data from studies without standardized protocols or techniques may have resulted in bias and yielded results that are difficult to interpret and translate to clinical settings. Third, the included studies provided limited data on lesion size, LM type, uterine sarcoma type, interreader variability in MRI reporting, and temporal parameters that can affect the diagnostic performance of MRI.

Despite current advances in imaging, there remains a lack of consensus regarding which MRI features are useful for differentiating LMS from LM. The results of our meta-analysis indicate that increased signal intensity on T1WI and low ADC values can provide accurate differentiation. Although, increased signal intensity on T2WI had higher sensitivity for LMS compared to leiomyomas (93% vs 54.5%, 93% vs 53.9%), there was no statistical significance. We recommend that prospective studies with larger cohorts be carried out to further improve the consensus on the significant MRI features of LMS and LM.

## References

[1] Parker WH. Etiology, symptomatology, and diagnosis of uterine myomas. Fertil Steril. 2007; 87(4): 725–36. DOI: 10.1016/j.fertnstert.2007.01.093 17430732

[2] Wu TI, Yen TC, Lai CH. Clinical presentation and diagnosis of uterine sarcoma, including imaging. Best Pract Res Clin Obstet Gynaecol. 2011; 25(6): 681–9. DOI: 10.1016/j.bpobgyn.2011.07.002 21816678

[3] Ando T, Kato H, Furui T, Morishige KI, Goshima S, Matsuo M. Uterine smooth muscle tumours with hyperintense area on T1 weighted images: Differentiation between leiomyosarcomas and leiomyomas. Br J Radiol. 2018; 91(1084): 20170767 DOI: 10.1259/bjr.20170767 29308922 PMC5966007

[4] Lakhman Y, Veeraraghavan H, Chaim J, et al. Differentiation of Uterine Leiomyosarcoma from Atypical Leiomyoma: Diagnostic Accuracy of Qualitative MR Imaging Features and Feasibility of Texture Analysis. Eur Radiol. 2017; 27(7): 2903–15. DOI: 10.1007/s00330-016-4623-9 27921159 PMC5459669

[5] Li HM, Liu J, Qiang JW, et al. Diffusion-Weighted Imaging for Differentiating Uterine Leiomyosarcoma From Degenerated Leiomyoma. J Comput Assist Tomogr. 2017; 41(4): 599–606. DOI: 10.1097/RCT.0000000000000565 27997438

[6] Lin G, Yang LY, Huang YT, et al. Comparison of the diagnostic accuracy of contrast-enhanced MRI and diffusion-weighted MRI in the differentiation between uterine leiomyosarcoma/smooth muscle tumor with uncertain malignant potential and benign leiomyoma. J Magn Reson Imaging. 2016; 43(2): 333–42. DOI: 10.1002/jmri.24998 26383110

[7] Rio G, Lima M, Gil R, Horta M, Cunha TM. T2 hyperintense myometrial tumors: Can MRI features differentiate leiomyomas from leiomyosarcomas? Abdom Radiol (NY). 2019; 44(10): 3388–97. DOI: 10.1007/s00261-019-02097-x 31250178

[8] Sato K, Yuasa N, Fujita M, Fukushima Y. Clinical application of diffusion-weighted imaging for preoperative differentiation between uterine leiomyoma and leiomyosarcoma. Am J Obstet Gynecol. 2014; 210(4): 368.e1–368.e8. DOI: 10.1016/j.ajog.2013.12.028 24368137

[9] Tamai K, Koyama T, Saga T, et al. The utility of diffusion-weighted MR imaging for differentiating uterine sarcomas from benign leiomyomas. Eur Radiol. 2008; 18(4): 723–30. DOI: 10.1007/s00330-007-0787-7 17929022

[10] Tanaka YO, Nishida M, Tsunoda H, Okamoto Y, Yoshikawa H. Smooth muscle tumors of uncertain malignant potential and leiomyosarcomas of the uterus: MR findings. J Magn Reson Imaging. 2004; 20(6): 998–1007. DOI: 10.1002/jmri.20207 15558559

[11] Valdes-Devesa V, Jimenez MDM, Sanz-Rosa D, et al. Preoperative diagnosis of atypical pelvic leiomyoma and sarcoma: The potential role of diffusion-weighted imaging. J Obstet Gynaecol. 2019; 39(1): 98–104. DOI: 10.1080/01443615.2018.1466110 30207503

[12] Namimoto T, Yamashita Y, Awai K, et al. Combined use of T2-weighted and diffusion-weighted 3-T MR imaging for differentiating uterine sarcomas from benign leiomyomas. Eur Radiol. 2009; 19(11): 2756–64. DOI: 10.1007/s00330-009-1471-x 19504102

[13] DerSimonian R, Laird N. Meta-analysis in clinical trials. Control Clin Trials. 1986; 7(3): 177–88. DOI: 10.1016/0197-2456(86)90046-2 3802833

[14] Santos P, Cunha TM. Uterine sarcomas: clinical presentation and MRI features. Diagn Interv Radiol. 2015; 21(1): 4–9. DOI: 10.5152/dir.2014.14053 25347940 PMC4463355

[15] Kaganov H, Ades A, Fraser DS. Preoperative magnetic resonance imaging diagnostic features of uterine leiomyosarcomas: a systematic review. Int J Technol Assess Health Care. 2018; 34(2): 172–9. DOI: 10.1017/S0266462318000168 29642961

[16] Bammer R. Basic principles of diffusion-weighted imaging. Eur J Radiol. 2003; 45(3): 169–84. DOI: 10.1016/S0720-048X(02)00303-0 12595101

[17] Fujii S, Kakite S, Nishihara K, et al. Diagnostic accuracy of diffusion-weighted imaging in differentiating benign from malignant ovarian lesions. J Magn Reson Imaging. 2008; 28(5): 1149–56. DOI: 10.1002/jmri.21575 18972356

[18] Whittaker CS, Coady A, Culver L, et al. Diffusion-weighted MR imaging of female pelvic tumors: A pictorial review. Radiographics. 2009; 29(3): 759–74; discussion 74–8. DOI: 10.1148/rg.293085130 19448114

[19] Ando T, Kato H, Furui T, et al. Uterine smooth muscle tumours with hyperintense area on T1 weighted images: Differentiation between leiomyosarcomas and leiomyomas. The British Journal of Radiology. 2018; 91(1084): 20170767 DOI: 10.1259/bjr.20170767 29308922 PMC5966007

